# Merged image reconstruction for anomalous systemic arterial supply to a normal lung

**DOI:** 10.1002/jmrs.383

**Published:** 2020-03-02

**Authors:** Bin Hu, Yunping Lan, Qiang Li, Xiaozun Yang, Bo Tian, Haomiao Qing, Peng Zhou, Ting Wang, Xiaojun Yang

**Affiliations:** ^1^ Department of Thoracic Surgery Sichuan Cancer Hospital and Institute, the affiated Cancer Hospital School of Medicine, University of Electronic Science and Technology of China Chengdu China; ^2^ Intensive Care Unit Sichuan Academy of Medical Sciences and Sichuan Provincial People's Hospital School of Medicine, University of Electronic Science and Technology of China Chengdu China; ^3^ Medical imaging Department Sichuan Cancer Hospital and Institute, the affiated Cancer Hospital School of Medicine, University of Electronic Science and Technology of China Chengdu China; ^4^ Sichuan Lung Cancer Institute West China Hospital Sichuan University Chengdu China

**Keywords:** Angiography, pulmonary artery hypoplasia, systemic arterial supply to the lung, three‐dimensional imaging, vascular disease

## Abstract

We present a rare case of anomalous systemic arterial supply to normal basal segments of the left lower lobe. Plain computed tomography (CT) showed an occupancy lesion in the left lower lobe. Contrast CT and merged three‐dimensional (3D) image reconstruction showed that the anomalous systemic artery originated from the descending aorta and substituted the basilar segmental pulmonary artery and the arterial supply to the basilar segment of left lower lobe. We use the merged image reconstruction of 3D CT angiography and bronchography (3D‐CTAB) to depict the precise location and stereoscopic shape of this vascular malformation. Therefore, we think that these data add a novel comprehensive perspective on the diagnosis of the feature of malformation and treatment planning for this rare disease.

## Graphical abstract

We present a rare case and images of anomalous systemic arterial supply to normal basal segments of the left lower lobe. We use the merged image reconstruction of three‐dimensional computed tomography angiography and bronchography to depict the precise location and stereoscopic shape of this vascular malformation. Therefore, these data add a novel comprehensive perspective on the diagnosis of the feature of malformation and treatment planning for this rare disease.

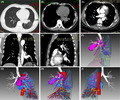

## Introduction

Anomalous systemic arterial supply to a normal lung (ASALL) is a rare congenital systemic pulmonary vascular malformation.^1^, ^2^, ^3^, ^4^ The anomalous systemic artery (ASA) can be located by digital subtraction angiography (DSA) and computed tomography (CT) angiography.^5^, ^6^, ^7^ A novel merged three‐dimensional (3D) image technique can be used to reconstruct and combine images obtained in angiography, bronchography and parenchyma together, and this approach has been extensively used in the identification of target bronchi and vessels, determining the location of the intersegmental plane and performing operative simulation of segmentectomy.^8^ Compared with previous image techniques, 3D CT angiography and bronchography (3D‐CTAB) allows the stereoscopic anatomy and feature of ASALL to be precisely evaluated.

## Case Report

**Figure 1:**
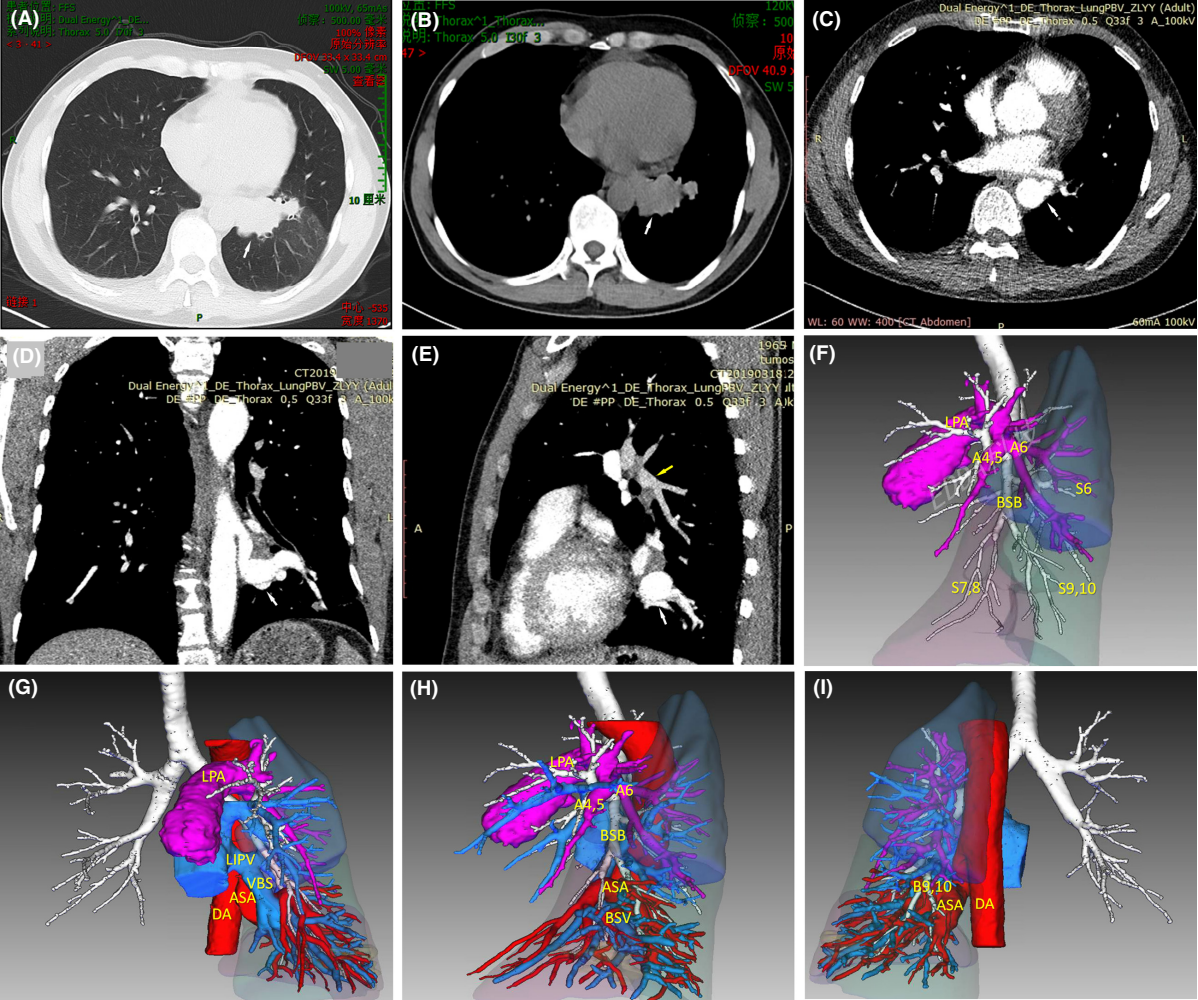
Computed tomography (CT)/CT angiography and three‐dimensional (3D) reconstruction depicting the anomalous systemic arterial (ASA) supply to the normal basal segments of the lung (A). (B) A plain CT showing a mass in the left lower lobe. Contrast‐enhanced CT angiography showing the origin and supply of the ASA (white arrow) in coronal (C), axial (D) and sagittal (E) views. The left pulmonary artery (PA) ended in the dorsal segment (yellow arrow). (F–I) Merged three‐dimensional reconstructed image used to locate the ASA (G) and (I), which substituted the PA basal segments (F) and (H) showed that the bronchi and pulmonary vein connected normally. A4,5, lingual artery segment; A6, dorsal artery segment; ASA, anomalous systemic arterial; BSB, basal bronchial segment; B9,10, bronchi of the lateral and posterior basal segments; DA, descending aorta; LIPV, left inferior pulmonary vein; LPA, left pulmonary artery; S6, dorsal segment; S7,8 median and anterior basal segment; S9,10 lateral and posterior basal segments; and VBS, basal vein segments.

A 53‐year‐old male patient was admitted with cough and blood in the phlegm and found to have an occupancy lesion (3.4 × 3.1 cm maximal section) in the left lower lobe on plain CT (Fig. 1A,B). The symptoms and mass images indicated lung neoplasm.

The patient had undergone a dual‐source CT scan (Definition, Siemens, Munich, Germany). The patient was scanned approximately 16–20 sec after contrast agent was injected. The scanning range was defined from the plane of the thoracic inlet to the diaphragmatic plane. The collimator thickness was 0.6 mm, and the reconstruction thickness was 1 mm.

CT angiography showed that the mass was an ASA originating from the descending aorta with a saccular aneurysm in the basal segments of the left lower lobe (Fig. 1C–E, Video S1). The patient was diagnosed with anomalous systemic arterial supply to normal basal segments of the left lower lobe.^1^ The distal left pulmonary artery ended and was distributed in the lingual and dorsal segments, while the pulmonary artery basal segments were absent and substituted by the ASA (Fig. 1E–G).

DICOM data were transferred to an intelligent/interactive qualitative and quantitative analysis (IQQA) 3D reconstruction system and its web based workstation (EDDA Technology, Princeton Junction, NJ, USA). The differences in the density of the contrast agent, as well as the measurements such as axis direction and vessel diameter, are automatically identified to distinguish between arteries and veins. The data are analysed, calculated, segmented, and rendered to describe and interpret the morphology and spatial position of the structures. And the IQQA 3D‐reconstructed images of vessels, bronchi and segments are merged into a single 3D image. The location and course of the ASA was identified (Fig. 1G, Video S2). Based on the course of the bronchi and blood vessels, the spatial conformation of the bronchial tree and accompanied arterial tree is reconstructed, and the stems of venous branches threading between are intersegmental veins, which manages the automated the lobulation, segmentation and subsegmentation (Fig. 1G–I, Video S2).^8^ Then, we had got a clear imaging profile of the disease. The patient recovered from cough and blood in the phlegm with medical treatment denied further invasive therapy to cure this disease although being informed high‐risk prognosis of conservative measures.

Permission was obtained from the patient to publish this case study.

## Discussion

ASALL can be differentiated from intrapulmonary sequestration (Pryce I), although both diseases have ASA, and the parenchyma and bronchi developed normally in this case without sequestration.^1^, ^2^, ^3^, ^4^ The pulmonary veins developed normally, while the ASA increased pressure on the pulmonary vascular bed, causing a left‐to‐left shunt mechanism to be embedded in and the left cardiac load and pulmonary capillaries to burst out blood. So this patient was admitted with cough and blood in the phlegm and an occupancy lesion in the lung for suspected tumor.^5^, ^6^

The DSA and reconstruction of CT angiography images allow the location of the lesion and its course. However, being subjected to transient developing and agent image phases, it cannot be effective in obtaining a simultaneous profile of adjacent structures and the vascular and bronchial distribution of the involved segments on those two‐dimensional images of DSA and CT.^5^, ^6^, ^7^, ^9^ The IQQA synthetic reconstructed images can be observed from all directions, allowing a better delineation of the pertinent structures, the precise range of anomalous artery‐supplied segments and the evaluation of pulmonary artery hypoplasia.^8^

Guided by the 3D image, we could simulate and design the interventional therapy of transarterial embolisation of ASA or surgical therapies.^10^, ^11^ The simple and direct surgical design could be lobectomy or vasectomy.^5^, ^6^, ^10^, ^11^, ^12^ Although suddenly obstruct the arterial supply to the basal segment by embolisation or vasectomy, there was no report of severe pulmonary infarction case.^10^, ^11^, ^12^ The design of ASA and pulmonary artery anastomosis aims to shunt the pressure of systemic circulation of the ASA to the aplastic pulmonary artery to reduce the volume of aneurysm and promote pulmonary angiogenesis.^13^ Thoracoscopic segmentectomy guided by 3D‐CTAB has been widely accepted in a number of institutes. Precise anatomic segmentectomy has minimised the unnecessary removal of healthy parenchyma. In our case, a precise division of ASA and basilar bronchus could reveal clear borders of the intersegmental plane between basal segments and dorsal segment, which are the surgical margin of the basal segments and the ASA supplied areas.^7^, ^8^ Therefore, IQQA 3D CT reconstructed images add a novel comprehensive perspective for the diagnosis of and treatment planning for this rare congenital vascular malformation.

## Supporting information


**Video S1.** Dynamic display CT angiography images.

**Video S2.** Reconstruction of CT image to locate and delineate pertinent structures.
